# Unusual Metastases in Renal Cell Carcinoma: A Single Institution Experience and Review of Literature

**DOI:** 10.4021/wjon232w

**Published:** 2010-08-29

**Authors:** Cynthia Villarreal-Garza, Sandra I. Perez-Alvarez, Ivan R. Gonzalez-Espinoza, Eucario Leon-Rodriguez

**Affiliations:** a Department of Hematology and Oncology, Instituto Nacional de Ciencias Medicas y Nutricion Salvador Zubiran, Mexico City, Mexico

**Keywords:** Renal cell carcinoma, Renal cancer, Unusual metastases, Atypical metastases, Single institution experience, Metastatic renal cancer

## Abstract

**Background:**

To report location and management of atypical metastases from renal cell carcinoma (RCC) in the Instituto Nacional de Ciencias Medicas e Investigacion Salvador Zubiran (INCMNSZ) in Mexico City.

**Methods:**

Between 1987 to 2009, 545 patients with RCC were retrospectively identified at the INCMNSZ. Patients with unusual metastases confirmed by histopathology were analyzed. Epidemiological, clinical, diagnosis, treatment and outcome data were reviewed.

**Results:**

Sixty patients developed 98 unusual metastases secondary to RCC. The group was comprised of 35 men (58.3%), with a median age of 60 years at diagnosis. Metachronous unusual metastases with primary renal cancer were observed in 37 individuals (61.7%). Median time from primary RCC diagnosis to the first unusual metastasis was 16.5 months. Median survival from diagnosis of the first unusual metastasis to death was 5.0 months (CI 95%: 2.8-7.2 months). Patients with an initial solitary metastatic lesion in an unusual site (28.3%) had a better survival compared to patients who primarily presented with multiple metastases, 17.0 (CI 95%: 6.1-27.9) Vs 3.0 months (CI 95%: 0.9-5.1), p = 0.001. Unusual metastasis resection (21 patients) improved survival, 25.0 (CI 95%: 5.1-44.9) Vs 3.0 months (CI 95%: 0.8-5.2), p < 0.0001. No survival difference was observed between localization of unsual metastases (p = 0.72).

**Conclusions:**

In patients with advanced RCC we suggest an individual diagnostic and surgical approach to achieve complete resection with disease-free margins, even in the presence of unusual metastatic sites, multifocality, or history of metastasectomy. These strategy might provide not only palliation for symptoms, but an opportunity for meaningful disease free and overall survival.

## Introduction

In 2005, worldwide, renal cell carcinoma (RCC) accounted for 208,000 new cases, 1.9% of all systemic malignancies and 102,000 deaths [1]. When compared with 1971, these numbers represent a fivefold increase in new cases and a twofold increase in mortality [2]. A quarter of patients with RCC present with advanced disease, including locally invasive or metastatic cancers. Moreover, a third of patients undergoing resection of localized disease will have recurrence [3]. RCC metastases can present many years after initial treatment, with recurrences reported up to 17.5 years after initial surgery.

The most common sites of metastatic involvement are lung parenchyma, bones, liver, and brain [4]. In addition, it has been reported that adrenal metastases from primary RCC are found in up to 10% of patients with advanced tumor stages [5]. On the other hand, unusual metastases have been described in RCC, and virtually any organ site can be involved. Although RCC is characterized with unpredictable clinical presentation and biologic behavior, infrequent localization of metastases is unexpected and mode of spread is obscure.

In the present study, we aimed to assess the incidence, clinical presentation, and surgical treatment in a cohort of patients with unusual metastatic lesions secondary to RCC treated in a single Mexican Institution. We also reviewed previous published reports of this entity and compare their results with our findings.

## Patients and Methods

All patients diagnosed with RCC between 1987 to 2009 at the Instituto Nacional de Ciencias Medicas y Nutricion Salvador Zubiran INCMNSZ in Mexico City were retrospectively identified (545 patients). Patient hospital records and office charts were reviewed. Patients diagnosed with unusual metastatic sites were recognized and selected for analysis. Common metastases including lung, liver, brain, and adrenal were excluded. Demographics, histology, stage, treatment, recurrence, and overall survival were analyzed.

The diagnosis of RCC in patients with unusual metastases that presented at the time of the primary RCC diagnosis was either done in the nephrectomy specimen or histologically confirmed on the unusual metastasis. Patients with infrequent metastases recognized together with other common-site metastases required pathological corroboration of any of the metastatic lesions. Those individuals diagnosed with a single metachronous metastasis required histological confirmation to be included in the analysis.

The time to progression was defined as the interval between curative nephrectomy and recurrence and the overall survival from curative nephrectomy to death. The time from the diagnosis of the initial unusual metastasis to death was calculated.

Continuous variables were expressed as median and standard deviation and categorical variables as percentage and 95% confidence intervals. Median recurrence-free survival (RFS) and overall survival (OS) were estimated by the Kaplan-Meier method, and the survival analyses were assessed by log-rang test. A value of *p* < 0.05 was considered significant (two-sided tails). All statistical computations were performed using the SPSS version 14.0 statistical software package (SPSS Inc, Chicago, IL).

Articles in the English language literature were systematically searched in Medline and PubMed with the combination of the following key words: renal cell carcinoma, renal cell cancer, kidney carcinoma, kidney cancer, with unusual or infrequent metastases to gastric, stomach, pancreas, duodenum, ampulla, ampullary, biliary tract, colon, spleen, splenic, peritoneal, peritoneum, ovary, ovarian, uterus, vagina, cervix, testicle, heart, eye, orbit, nose, muscle, diaphragm, ligament, skin, cutaneous, and thyroid. Individual case reports, cases series of metastatic RCC, as well as other reviews detailing lists of infrequent RCC metastases were reviewed. Cross-references from these articles not highlighted in the Medline or Pubmed search were also obtained and reviewed.

## Results

**Figure 1:**
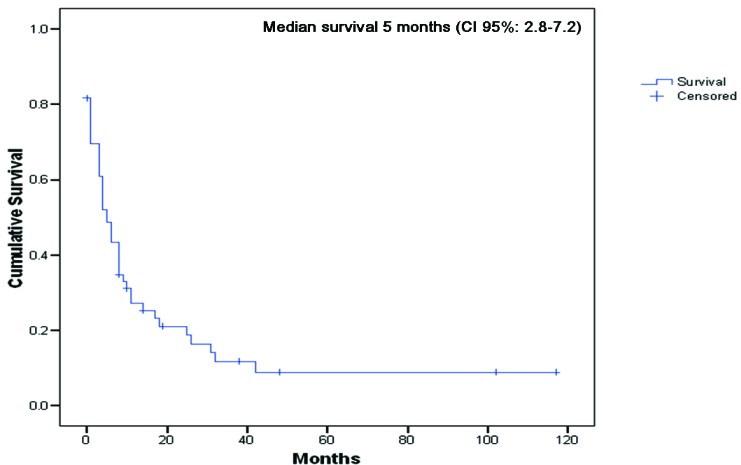
Overall survival from diagnosis of the first unusual metastasis to death or last follow-up (n = 60).

Sixty patients were identified who developed 98 unusual metastases secondary to renal carcinoma. The group was comprised of 35 men (58.3%) and 25 women (41.7%), with a median age of 60 years, standard deviation (SD) 11.5, at the time of diagnosis of unusual metastases. Thirty-eight patients (63.3%) had a positive smoking history. Synchronous unusual metastases with primary renal cancer were observed in 23 patients (38.3%), whereas metachronous metastases were found in 37 individuals (61.7%). The median time from primary renal cancer diagnosis to the first unusual metastasis was 16.5 months (range: 0-300 months). Median overall survival from primary cancer diagnosis was 31.0 months (CI 95%: 11.2-50.8). Additionally, the median survival from diagnosis of the first unusual metastasis to death was 5.0 months (CI 95%: 2.8 - 7.2 months), Figure 1.

The characteristics of the 98 unusual metastases are summarized in Table 1. These included 23 patients with metastases in skin, 15 muscle, 12 colon, 11 peritoneum, 11 pancreas, 5 spleen, 4 duodenum, 4 stomach, 2 biliary tract, 2 ligaments, 2 thyroid, 1 esophagus, 1 small intestine, 1 vagina, 1 ovary, 1 testicle, 1 heart, and 1 periuretral tissue. The muscle metastases were distributed as follows: 4 paravertebral, 4 diaphragm, 3 psoas, 1 transversus abdominis, 1 gluteus maximus, 1 quadratus lumborum, and 1 inferior rectus extraocular metastasis. The ligaments involved by renal cell cancer were the uterus posterior ligament and falciform ligament.

**Table 1:** Patient Demographic, Clinical and Outcome Characteristics

Site of metastases	Number (%)	Gender (M:F)	Age (Median) (Mean) (Range)	Months from diagnosis to unsual metastases (Median) (Mean) (Range)	Synchronous metachronous (S:M)	Histology	Unusual localization as initial unique site of metastases	Status (Alive:Dead)	Palliative nephrectomy Total (%)	Surgical resection of metastases Total (%)	Months from diagnosis to death or last follow up (Median in months) (Range)	Months from diagnosis of unsual metastasis to death or last follow up (Median in months) (Range)
Skin	23 (23.46%)	16:7	54 54.2 (19-70)	7 14.3 (0-73)	11:12	Clear: 22 Papillary: 1	4 (17.4%)	2 (8.7%) 21 (91.3%)	6 (26.1%)	6 (26.1%)	11 0-76	4 0-42
Muscle	15 (15.31%)	6:9	57 56.4 (46-66)	14 18.27 (1-83)	3: 12	Clear: 14 Papillary: 1	1 (6.6%)	4 (26.7%) 11 (73.3%)	2 (13.3%)	2 (13.3%)	16 (1-74)	1 (0-32)
Colon	12 (12.24%)	6:6	63 62.3 (37-84)	11 36.4 (0-128)	4:8	Clear: 12	2 (16.7%)	2 (16.7%) 10 (83.3%)	3 (25.0%)	4 (33.3%)	12.5 (1-146)	1 (0-25)
Peritoneal metastases	11 (11.22%)	8:3	61 54.64 (19-69)	43 57.91 (0-195)	2: 9	Clear: 10 Papillary: 1	5 (45.5%)	2 (18.2%) 9 (81.8%)	2 (18.2%)	3 (27.3%)	38 (1-196)	0 (0-38)
Pancreas	11 (11.22%)	7:4	55 55.6 (42-68)	97 99.27 (0-300)	4:7	Clear: 11	3 (27.3%)	3 (27.3%) 8 (72.8%)	1 (9.1%)	3 (27.3%)	106 (2-351)	6 (1-102)
Spleen	5 (5.10%)	3:2	64 65.2 (48-84)	7 32.2 (0-86)	2:3	Clear: 5	3 (60%)	1 (20%) 4 (80%)	1 (20%)	1 (20%)	16 (1-106)	6 (0-19)
Duodenum	4 (4.08%)	3:1	50.5 50.8 (43-59)	88.5 93.0 (6-189)	1:3	Clear: 4	2 (50%)	0 (0%) 4 (100%)	1 (25%)	0 (0%)	91 (7-195)	1.5 (0-6)
Stomach	4 (4.08%)	0: 4	63 66.75 (57-84)	2 15 (0-56)	3: 1	Clear: 4	0 (0%)	2 (50%) 2 (50%)	2 (50%)	1 (25%)	3 (0-76)	0.5 (0-19)
Biliary tract	2 (2.04%)	1:1	61 61 (58-64)	207 207 (119-295)	0:2	Clear: 2	1 (50%)	2 (100%) 0 (0%)	0 (0%)	1 (50%)	231 (167-295)	24 (0-48)
Ligaments	2 (2.04%)	1:1	58.5 58.5 (53-64)	30.5 30.5 (18-43)	0: 2	Clear: 2	1 (50%)	0 (0%) 2 (100%)	0 (0%)	0 (0%)	39.5 (36-43)	9 (0-18)
Thyroid	2 (2.04%)	0:2	49 49 (40-58)	84 84 (79-89)	0:2	Clear: 2	2 (100%)	1 (50%) 1 (50%)	0 (0%)	1 (50%)	151.5 (107-196)	67.5 (18-117)
Esophagus	1 (1.02%)	0:1	55	60	0:1	Clear: 1	1 (100%)	0 (0%) 1 (100%)	0 (0%)	1 (100%)	71	11
Small intestine	1 (1.02%)	1:0	54	0	1:0	Clear: 1	0 (0%)	0 (0%) 1 (100%)	1 (100%)	1 (100%)	3	3
Vagina	1 (1.02%)	0:1	49	10	0: 1	Clear: 1	0 (0%)	0 (0%) 1 (100%)	0 (0%)	0 (0%)	19	8
Ovary	1 (1.02%)	0: 1	64	7	0: 1	Clear: 1	1 (100%)	1 (100%) 0 (0%)	0 (0%)	1 (100%)	16	8
Testicle	1 (1.02%)	1: 0	48	33	0: 1	Clear: 1	0 (0%)	0 (0%) 1 (100%)	0 (0%)	1 (100%)	48	15
Heart	1 (1.02%)	1:0	47	34	0: 1	Clear: 1	0 (0%)	0 (0%) 1 (100%)	0 (0%)	0 (0%)	35	0
Periuretral tissue	1 (1.02%)	0: 1	65	45	0: 1	Clear: 1	0 (0%)	0 (0%) 1 (100%)	0 (0%)	1 (100%)	54	8
Total	98 (100%)	54:44	58 56.81 (19-84)	15 42.79 (0-300)	31:67	Clear: 95 Papillary: 3	26 (26.5%)	20 (20.5%) 78 (79.6%)	19 (19.4%)	27 (27.6%)	23.5 (0-351)	3 (0-117)

**Figure 2:**
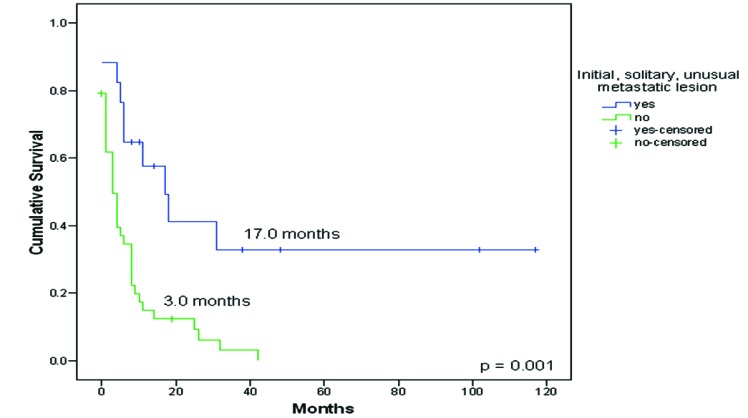
Survival from diagnosis of patients with an initial solitary unusual metastatic lesion to death or last follow-up (n = 17) compared to patients who primarily presented with multiple metastases (typical or atypical localizations) (n = 43).

Seventeen patients (28.3%) presented initially with a solitary metastatic lesion in an unusual site. These patients had a better survival compared to patients who primarily presented with multiple metastases (typical or atypical localizations), 17.0 months (CI 95%: 6.1-27.9) Vs 3.0 months (CI 95%: 0.9-5.1), p = 0.001, Figure 2. No statistical difference in survival was encountered between synchronous and metachronous metastases (p = 0.37). No difference in survival was observed between patients with disease-free interval longer or shorter than one year.

**Figure 3:**
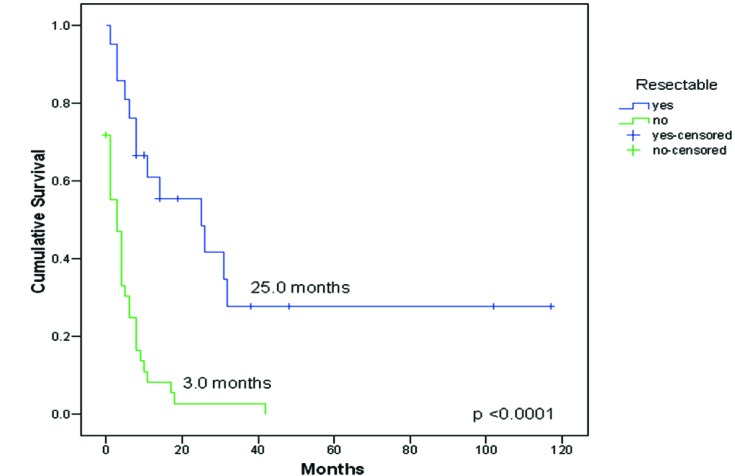
Survival from diagnosis of the first unusual metastasis to death or last follow-up compared between resectable (n = 21) vs no-resectable (n = 39) metastasis.

Twenty-one patients had complete resections of their unusual metastases: in 12 the unusual metastatic site was diagnosed as the initial and solitary metastatic lesion (before appearance of any other common metastasis), and in other 9 the unusual metastasis was removed after initial surgical treatment of other common metastases. Unusual metastasis resection improved survival, 25.0 months (CI 95%: 5.1-44.9) Vs 3.0 months (CI 95%: 0.8-5.2), p < 0.0001, Figure 3.

Three patients had multiple complete resections of unusual metastatic lesions that were diagnosed in a sequential form. The first patient was treated with resection of unusual metastases in the spleen and ovary seven months after primary renal cancer diagnosis. Following seven more months, colon cancer metastasis and a cutaneous nodule were resected. No palliative systemic treatment was administered. The patient is still alive 16 months since renal cancer diagnosis and 8 months after resection of the first two metastases.

In the second patient, metastatic resection of contralateral adrenal metastasis and a pulmonary lesion were done 32 and 47 months after primary cancer diagnosis, respectively. Afterwards, unusual metastasis in stomach and colon were resected, 56 and 60 months after primary renal cancer diagnosis, respectively. The patient received systemic treatment with interferon. After 13 more months, new multiple metastatic lesions were identified in pancreas and cutaneous nodules. The patient died 76 months after primary renal cancer diagnosis, 44 months after resection of first resected metastases and after 20 months of first resected unusual metastasis.

The last patient had resection of a diaphragm metastasis 17 months after renal cancer diagnosis. He was initially treated with thalidomide. Sixteen months later, he was diagnosed with testicular metastasis that was surgically removed, and was treated with interferon. Twelve months after, he developed pulmonary and cerebral metastatic lesions. He died 48 and 31 months after primary renal cancer diagnosis and after first unusual metastasis, respectively.

**Figure 4:**
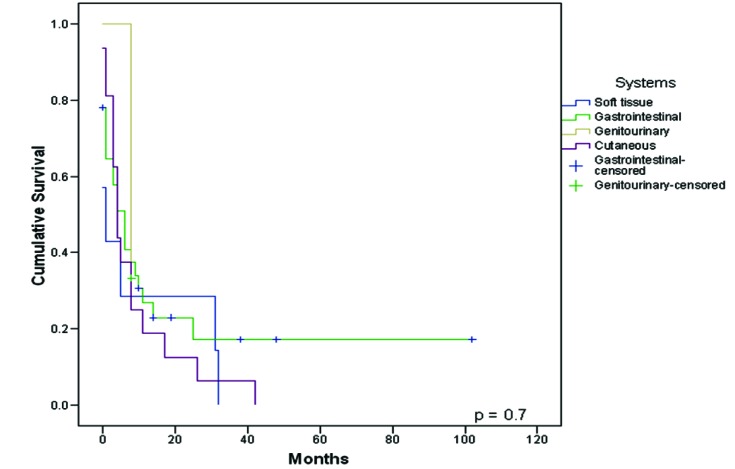
Survival from diagnosis of the first unusual metastasis to death or last follow-up compared between systems.

When differences in survival between localization were analyzed (cutaneous Vs soft tissue Vs gastrointestinal Vs genitourinary metastases), no statistical difference was observed among the groups (p = 0.72), Figure 4.

## Discussion

To our knowledge, this is the largest single institution experience that analyses unusual metastatic sites of RCC and its outcome dependent on presentation and surgical treatment. We retrospectively identified 545 patients with RCC diagnosis, in which 60 patients (11%) were diagnosed with 98 unusual metastates.

The most frequent unusual metastases were of gastrointestinal, cutaneous, and muscular location (Table 2). In contrast to the total number of cases in the literatature review, we found that gastrointestinal metastases occurred most frequently (51% vs 37%), and that the most common localization was large intestine (12% vs 3%), pancreas (11% vs 28%) and peritoneum (11% vs 0.8%). Additionally, in this series the head and neck metastases were not as frequently described as previous reports (2% vs 37%). Interestingly, cutaneous and muscle metastastic sites were frequently found in this report (23% vs 6% and 15% vs 4%, respectively).

**Table 2:** Unusual Metastases From Renal Cell Carcinoma: Cases Reported In The Literature.

Localization of unusual metastases	References	Number of cases reported (%)	Number of cases at our institution (%)
GASTROINTESTINAL		369 (36.9%)	50 (51.02%)
Pancreas	17-131	282 (28.2%)	11 (11.22%)
Large intestine	132-157	27 (2.7%)	12 (12.24%)
Stomach	158-176	23 (2.3%)	4 (4.08%)
Duodenum/Ampulla of Vater	177-190	16 (1.6%)	4 (4.08%)
Peritoneum	191-196	8 (0.8%)	11 (11.22%)
Biliary tract	197-203	7 (0.7%)	2 (2.04%)
Spleen	204-207	6 (0.6%)	5 (5.10%)
Esophagus	208-209	2 (0.2%)	1 (1.02%)
HEAD AND NECK		373 (37.3%)	2 (2.04%)
Thyroid	210-262	270 (27.0%)	2 (2.04%)
Eye and orbit	263-301	72 (7.2%)	0 (0%)
Nose and paranasal sinuses	302-315	31 (3.1%)	0 (0%)
SKIN	316-354	63 (6.3%)	23 (23.46%)
GENITOURINARY		55 (5.5%)	3 (3.06%)
Uterus, vagina and cervix	355-371	24 (2.4%)	1 (1.02%)
Ovary	372-385	16 (1.6%)	1 (1.02%)
Testicle	386-396	15 (1.5%)	1 (1.02%)
MUSCLE		35 (3.5%)	15 (15.31%)
Skeletal	397-413	16 (1.6%)	5 (5.10%)
Diaphragm	414-416	6 (0.6%)	4 (4.08%)
Inferior rectus	417-421	5 (0.5%)	1 (1.02%)
Psoas	422-426	5 (0.5%)	3 (3.06%)
Gluteus maximus	427-428	2 (0.2%)	1 (1.02%)
Transversus abdominis	429	1 (0.1%)	1 (1.02%)
HEART	430-440	12 (1.2%)	1 (1.02%)
LIGAMENT		1 (0.1%)	2 (2.04%)
Falciform ligament	441	1 (0.1%)	2 (2.04%)
TOTAL		1000 (100%)	98 (100%)

Other publications have reported that solitary recurrences represent 55.7% in cases of typical sites of metastases [6]. In our series, 17 patients (28.3%) presented initially with a solitary metastatic lesion in an unusual site, in which 12 were amenable to resection.

The median time to first recurrence after nephrectomy varies widely between series. For common metastatic sites the median time to recurrence reported is approximately 25 months [6]. Conversely, metastatic RCC with pancreatic, duodenum, and ampulla of Vater localizations typically occur after a longer period following initial nephrectomy, with a median interval ranging from 6.5 to 12 years [7], the longest inverval being 32 years [8]. In our study, the median time from primary renal cancer diagnosis to the first unusual metastasis was 16.5 months, ranging from 0-300 months. However, specifically in patients with pancreatic and duodenum metastases the time to progression was longer (88.5 to 97 months).

No difference in survival was encountered between patients with disease-free interval longer or shorter than one year, contrasting the results from Kavolius in their series of 278 patients with recurrent RCC presenting with typical metastases in lung, bone, nodal recurrence and brain, which described a longer free survival time as a good prognostic factor [6]. Thus, it is imperative to remain vigilant in post-nephrectomy patients upon presentation of new clinical symptoms.

The median survival for patients with stage IV disease (T4 primary tumor, N2 involvement, or distant metastases) is only 4 to 20 months in contemporary reports, and the five-year survival rate is less than 10 percent for patients with distant metastases [9, 10]. According to our results, the presence of unusual metastatic sites confers a poor prognosis with a median survival from diagnosis of the first unusual metastasis to death of 5 months.

Surgical resection of primary RCC and metastatic deposits remain the most effective treatment since chemotherapy, radiotherapy and hormonal therapy have generally proved ineffective. The resection of a single or limited number of metastases is not uncommon either in combination with nephrectomy or at subsequent relapse. The reported outcomes after resection have been variable, with five-year survival rates in small series ranging from 13 to 50 percent. The potential role of surgery is illustrated by the results from a series of 278 patients with recurrent RCC in common sites [6], in which 51 percent underwent removal of all of their metastatic disease with curative intent, 25 percent underwent partial resection of their metastatic disease, and 24 percent were treated without surgery. The most common resected metastatic sites of typical localization included lung, brain, bone, and soft tissue. The five-year overall survival rate was highest in patients treated with curative intent metastasectomy (44 versus 14 versus 11 percent, respectively). Survival rates after complete resection of a second and third metastasis were not different from those after initial metastasectomy. In the same manner, in our experience we found that the complete resection of unusual metastatic sites confers a survival advantage, reaching a median survival time of 25 months compared to 3 months in those patients without surgical management (five-year survival of 30 vs 0%, respectively). Other authors have reported promising five-year survival rates after resection of solitary unusual metastases, specifically in pancreas, duodenum and ampulla of Vater, ranging from 43 to 88% [11-13], as well as in thyroid metastases with survival rate of 36% at 9 years of follow-up [14].

Multifocality of metastases from RCC is not unusual; it ranges from 20% to 45% in different reports [7]. However as long as a complete resection is accomplished, outcome does not differ between patients with multiple metastases compared to solitary metastases, as described in a series of 187 cases of pancreatic metastases from RCC in which information about multifocality was available [15].

There are several reports suggesting that previously resected renal cell carcinoma metastasis at other sites including the thyroid, adrenal glands and lung should not discourage aggressive treatment of a secondary metastasis once it is confirmed to be the only site of recurrent disease [7, 16]. It has been recommended that previous disease recurrences, regardless of site, should not dissuade the surgeon from considering resection of metastases if patients are otherwise free of disease at the time of observation. We found three patients with sequential complete resections of unusual metastatic lesions, which achieved a long-term survival that reached between 16 and 76 months after primary renal cancer diagnosis.

In case of this report, we did not evaluate the other systemic therapies used for metastatic patients, since they varied significantly and almost did not include recent approved antitarget therapies, such as sunitib, sorafenib, temsirolimus, or bevacizumab. Others have suggested that surgical resection combined with systemic treatment strategies might generate synergistic antitumor activity with improvement in outcomes [7]. How to combine surgery with medical treatment in metastatic RCC will be an important field of investigation in the near future.

In conclusion, in patients with advanced RCC we suggest an individual diagnostic and surgical approach to achieve complete resection with disease-free margins, even in the presence of unusual metastatic sites, multifocality, or history of metastasectomy. These strategy might provide not only palliation for symptoms, but an opportunity for meaningful disease free and overall survival.
